# Statistical design approach enables optimised mechanical lysis for enhanced long-read soil metagenomics

**DOI:** 10.1038/s41598-024-80584-y

**Published:** 2024-11-22

**Authors:** Daniel G. Barber, Harry T. Child, Gabrielle R. Joslin, Lucy Wierzbicki, Richard K. Tennant

**Affiliations:** Faculty of Environment, Science and Economy, Amory Building, Rennes Drive, Exeter, Devon, EX4 4RJ UK

**Keywords:** Long-read sequencing, Design of experiment, DNA purification, Metagenomics, Metagenome assembly, Cell lysis, Metagenomics, Microbial ecology

## Abstract

Metagenomic analysis has enabled insights into soil community structure and dynamics. Long-read sequencing for metagenomics can enhance microbial ecology by improving taxonomic classification, genome assembly, and functional annotation. However, protocols for purifying high-molecular weight DNA from soil are not yet optimised. We used a statistical design of experiments approach to enhance mechanical lysis of soil samples, increasing the length of purified DNA fragments. Low energy input into mechanical lysis improved DNA integrity, resulting in longer sequenced reads. Our optimized settings of 4 m s^−1^ for 10 s increased fragment length by 70% compared to the manufacturer’s recommendations. Longer reads from low intensity lysis produced longer contiguous sequences after assembly, potentially improving a range of down-stream analyses. Importantly, there was minimal bias exhibited in the microbial community composition due to lysis efficiency variations. We therefore propose a framework for improving the fragment lengths of DNA purified from diverse soil types, improving soil science research with long-read sequencing.

**Supplementary Information:**

The online version contains supplementary material available at 10.1038/s41598-024-80584-y.

## Introduction

The microbial communities found in soil are some of the most diverse and complex of any ecological system^1^. Understanding the diversity and function of the observed soil microbiome is fundamental to interpreting the interconnected functional groups of Prokaryote, Eukaryote, Viral and Archaeal species within soils^2^. Increasingly, whole genome sequencing (WGS) is used as a method for analysing community function instead of computationally inferring the presence of gene pathways from metabarcoding data^3^. The large number of uncharacterised microbes present in soil demonstrates the necessity for using WGS to accurately determine soil microbial functionality^4^. It has also been suggested that many differences between soil community assemblages characterised to the species level, may mask marked similarity in the structure of metabolic function within those communities^5^. Community function analysis requires functional annotation of identified genes^6^ and often the generation of metagenome assembled genomes (MAGs) using WGS data^7^. Both the classification and subsequent functional annotation of sequence reads are improved by long-read sequencing technology^8^, driven by the now comparable error rates between long-read and short-read DNA sequencing^9^. It is also well documented that long-read sequence data greatly improves genome assembly especially in resolving long repeating and homopolymer regions of the genome^10^.

Obtaining high-quality, high-molecular weight DNA from the soil matrix is key to maximising the benefits of long-read sequencing. Nucleic acid extraction from soil typically involves mechanical lysis of the microbes within the sample when using commercially available extraction kits designed for use with soil samples^11,12^. Mechanical lysis enhances DNA yield and representation of resistant cell types, but reduces DNA integrity^13–15^. Although bench top homogenisers are typically used for this step in the laboratory, hand-held homogenisation devices can be used in the field^16^. The field application of DNA purification protocols can mitigate the effect of storage on the identified microbiome^17^, enabling sampling at sites that pose logistical challenges^18^. Optimisation of the mechanical lysis step to obtain higher molecular weight DNA is not explicitly detailed in soil DNA extraction protocols from commercial kits, as they are not designed for exclusive use with long-read sequencing technology. Systematic manipulation of mechanical lysis parameters to increase the length of DNA fragments purified from soil would improve multiple downstream analyses. It is important to maintain sufficient DNA yield for sequencing library preparation and ensure that the microbial diversity captured in subsequent data is not adversely affected in the pursuit of longer sequence reads.

Here we implement a statistical design of experiment (DoE) approach to optimise the mechanical lysis step of soil DNA extraction protocols both in the laboratory and the field. Maximising DNA fragment length and yield whilst maintaining representation of the full microbial diversity within the soil sample.

## Results

### Reduced homogenisation intensity increases DNA length without excessive yield reductions

A custom experimental design was generated to assess the main effects and 2nd order interactions of homogenisation speed, total homogenisation time and number of repeated homogenisation cycles for the mechanical lysis step of DNA purification from soil (Supplementary Table 1). Maximising DNA yield and mean DNA fragment length were specified as response variables for the design. Results from a scoping trial (Supplementary Table 2) were used to estimate root mean square error (RMSE) based on the standard deviation between replicate DNA extractions for both DNA yield and mean fragment length, expected effect sizes were also estimated from this data set. Power analysis confirmed (Supplementary Fig. 2) that the custom design had sufficient experimental runs and combination of parameters to reject the null hypothesis correctly > 90% of the time.

**Fig. 1:**
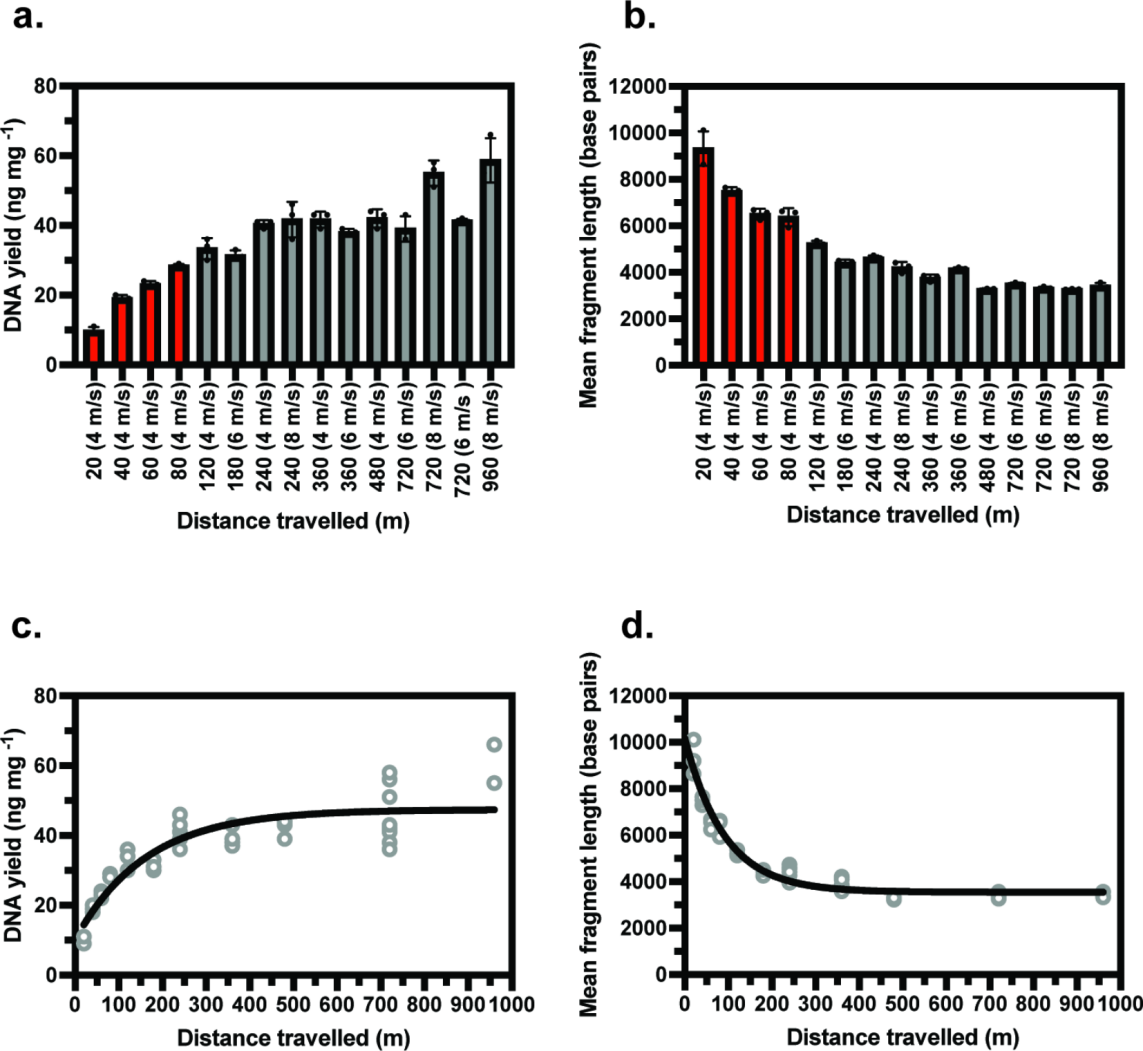
Yield and mean fragment length results from Custom DoE design. DNA yield (**A**) and mean fragment length (**B**) for arable soil DNA extractions conducted as part of the DoE custom design. Red bars indicate additional experimental runs conducted after evaluation of the least squares regression model. Statistical analysis displayed a positive relationship (Exponential plateau R^2^ 0.79) between DNA yield and increasing distance travelled by the homogenisation tube (**C**). A negative relationship (One phase decay R^2^ 0.96) was observed for mean fragment length and increasing distance travelled by the homogenisation tube (**D**). *n* = 3, error bars represent SD.

Based on the power analysis, the custom experimental design was used to investigate the impact of different mechanical lysis parameters on replicate DNA extractions performed on an arable soil sample. DNA yield and mean fragment length were quantified for each sample (Fig. 1). A standard least squares regression model was used to evaluate the contribution of each factor to the response variables based on the data generated from each DNA extraction. The number of repeat homogenisation cycles after resting on ice did not significantly contribute (p = ≥ 0.05) to changes in DNA yield or mean fragment length and so was removed from the model. Homogenisation speed, total homogenisation time and the 2nd order interaction between them were found to be significant factors in dictating both DNA yield and mean fragment length. A clear relationship between mean fragment length, homogenisation speed and time was observed, with the slowest speed and shortest duration homogenisation predicted to give the highest mean fragment length (Supplementary Fig. 3C). To visualise this overall trend, distance travelled by the homogenisation tube was calculated as a value that encompasses both speed and homogenisation time (Fig. 1C and D).

Results from the initial experimental design showed DNA yield was sufficient to prepare multiple sequencing libraries from all homogenisation conditions, with the lowest DNA yield of 80 ng µl^− 1^ in 100 µl. As DNA yield was not limiting, further runs were undertaken with shorter homogenisation time at 4 m s^− 1^ to investigate if mean fragment length could be further increased (Fig. 1A and B, red bars). An increase in mean fragment length was observed for shorter homogenisation time, reaching a maximum of 9,324 bp at 4 m s^− 1^ for 5 s (20 m distance travelled), more than double the 4,406 bp observed from lysis parameters often used in soil DNA extraction (180 m distance travelled, 6 m s^− 1^ for 30 s). DNA yield displayed a downward trend with shorter homogenisation time, although the lowest value of 10 ng mg^− 1^ of soil (or 2.5 µg total DNA) still represents sufficient yield for sequencing library preparation requirements using Oxford Nanopore Technology (ONT). An exponential plateau was fit to the DNA yield measurements indicating that DNA yield did not meaningfully increase beyond 300 m distance travelled for the purposes of input into sequencing library preparation (Fig. 1C, R^2^ 0.79). The opposite trend was observed for mean fragment length; a simple one phase decay model indicated DNA fragmentation did not increase beyond 400 m of travel by the homogenisation tube (Fig. 1D, R^2^ 0.96).

### Impact of homogenisation conditions is consistent across diverse soil types

To determine whether the results of the custom design were robust across different soil types, a full factorial design fixing homogenisation speed at 4 m s^− 1^ and constraining homogenisation time to 5,10,15 and 30 s was implemented. DNA extractions were performed on a pasture, heathland, and woodland soil (Supplementary Fig. S4). While absolute values differed between soil types, with the maximum mean fragment length exceeding 10,000 bp for the heathland and woodland soil compared to 7,982 bp for the pasture soil, the trend of increased homogenisation time reducing mean fragment length remained clear across the different soil types. Despite differences in absolute values, DNA yield increased with homogenisation time across the soil types with the minimum yield observed across all conditions (1.5 µg total DNA) remaining adequate for sequencing library preparation.

### Optimising lysis parameters for a hand-held homogeniser

**Fig. 2:**
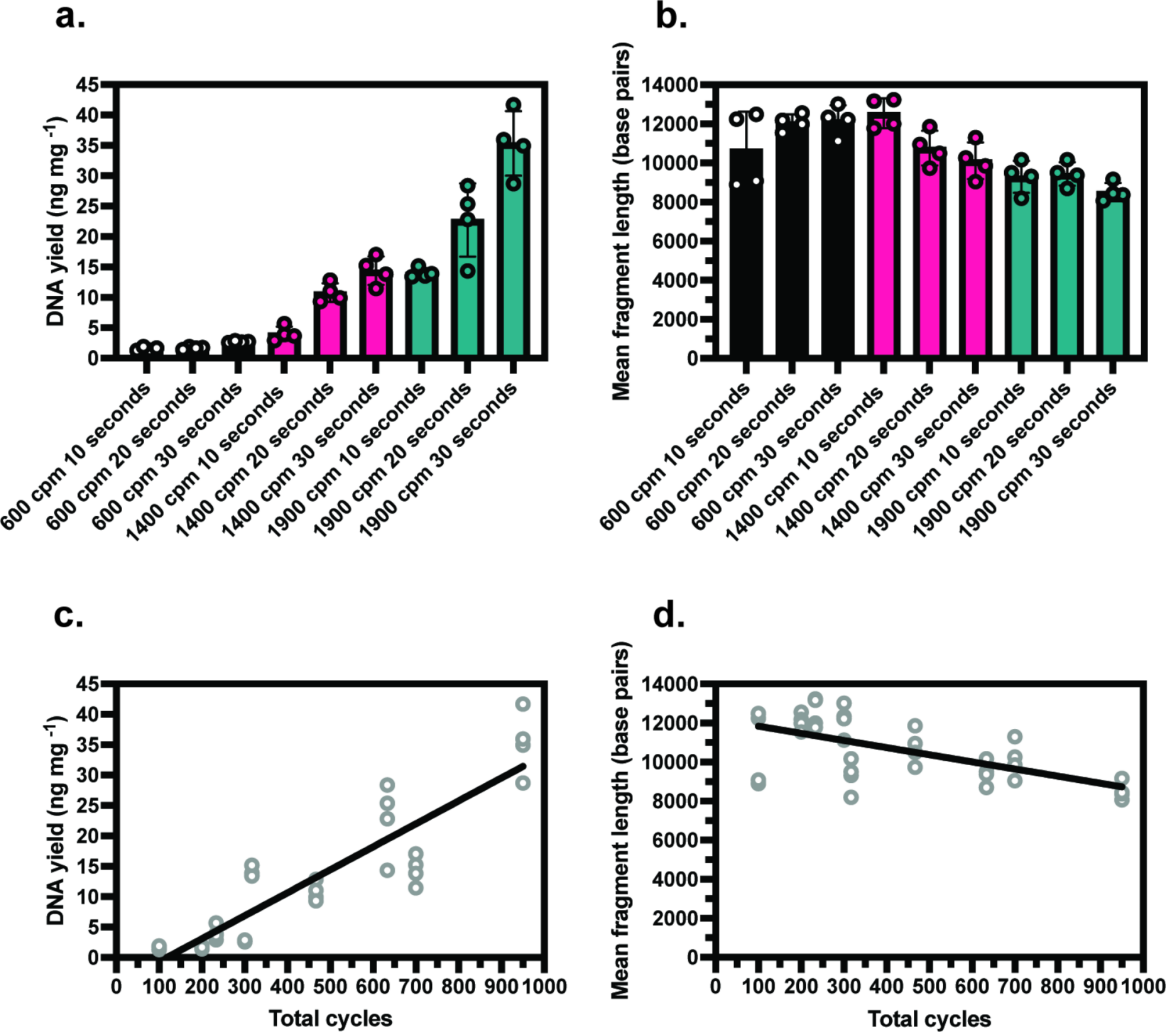
DNA yield and fragment length results using hand-held homogeniser. DNA yield (**A**) and mean fragment length (**B**) were measured for DNA extractions performed using the hand-held SuperFastPrep-2™ on heathland soil. Total cycles was calculated and linear regression analysis performed for DNA yield (**C**, R^2^ 0.81) and mean fragment length (**D**, R^2^ 0.37). *n* = 4, error bars represent SD.

Hand-held homogenisers offer the opportunity to perform DNA extractions on soil samples in the field. To compare the performance of hand-held and benchtop homogenisation, DNA was extracted from heathland soil using the SuperFastPrep-2™ (Fig. 2) with equivalent extractions performed using the FastPrep24-5G™ (Supplementary Fig. 5). DNA yield and mean fragment length was quantified for each extraction (Fig. 2A and B). To combine homogenisation speed and time into a single variable, total cycles were calculated for each sample as cycles per second multiplied by lysis time (Fig. 2C-D). Similar trends to the benchtop homogeniser were observed for both DNA yield and average fragment length, with DNA yield increasing alongside total cycles (Fig. 2C, R^2^ 0.81) and mean fragment length decreasing (Fig. 2D, R^2^ 0.34). The effect of total cycles on DNA yield was considerable, leading to concentrations below the input requirements for sequencing library preparation using the SQK-NBD.11,496 kit at lower homogenisation intensities. The effect on the mean fragment length was relatively minor, with the highest total cycles giving the lowest mean fragment length of 8,509 bp compared to 12,546 bp at 233 total cycles. In this comparison, the lowest mean fragment length of 8,509 bp observed in extractions using the hand-held homogeniser were comparable to the highest mean fragment length of 8791 bp for equivalent extractions using the benchtop homogeniser (Supplementary Fig. 5B).

### Reduced homogenisation intensity increases the length of DNA sequencing reads

**Fig. 3:**
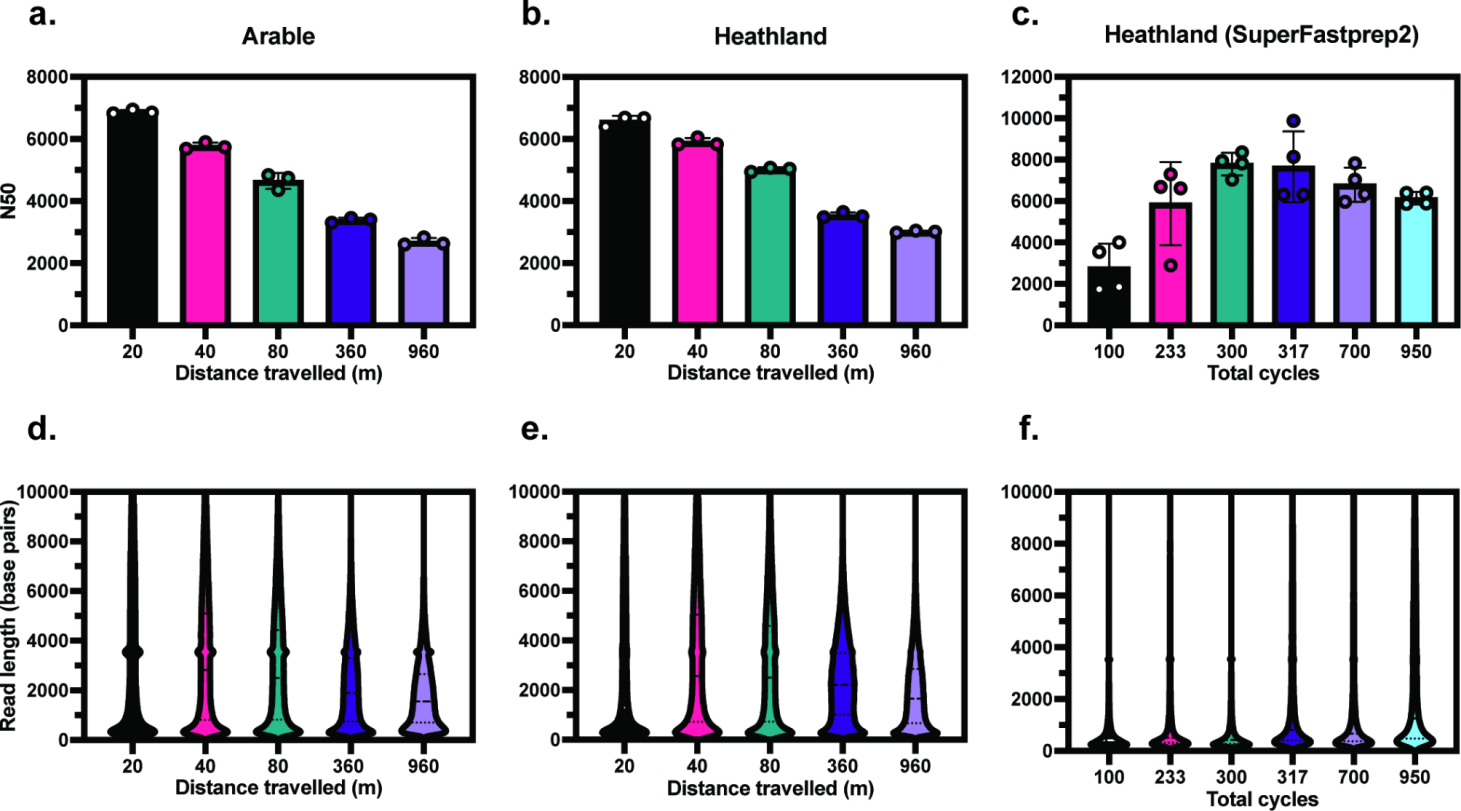
Sequencing metrics for different lysis parameters. N50 (**A**–**C**) and read length distribution (**D**–**F**) were quantified for each soil type after sequencing. *n* = 3 (**A**, **B**,**D**, **E**); *n* = 4 (**C**, **F**). Error bars represent SD. A one-way ANOVA detected significant differences in N50 between treatments (*p* < 0.05) for the arable soil (**A**. Kruskal-Wallis tests detected significant differences in N50 between treatments (*p* < 0.05) for both Heathland soil DNA extracted using the FastPrep-24™ 5G (**B**) and SuperFastPrep-2™ (**C**).

DNA extracts from the arable soil were selected for sequencing from groups of samples that had significantly different mean fragment lengths; 20 m (9,324 bps), 40 m (7,487 bps), 80 m (6,375 bps), 360 m (4,156 bps) and 960 m (3,418 bps) (one-way ANOVA, Tukey’s; pairwise comparison *p* < 0.05). These lysis parameters were then used to perform replicate extractions on heathland soil samples (Supplementary Fig. 5). Three replicates from each lysis condition were prepared for sequencing using the ONT Native Barcoding Kit and multiplexed on one MinION flow cell per soil type. N50 and read length distribution were quantified for each sample (Fig. 3). N50 decreased in agreement with the mean fragment length quantified for the raw extracts (Fig. 3A-B) with significant differences in N50 observed between lysis treatments for each group (one-way ANOVA, Kruskal-Wallis *p* < 0.05). DNA samples covering the full range of yield and fragment lengths measured for heathland soil extractions using the SuperFastPrep-2™ were also selected for sequencing (Fig. 3C) with a significant difference between lysis conditions detected (Kruskal-Wallis *p* < 0.05).

### Community analysis

**Fig. 4:**
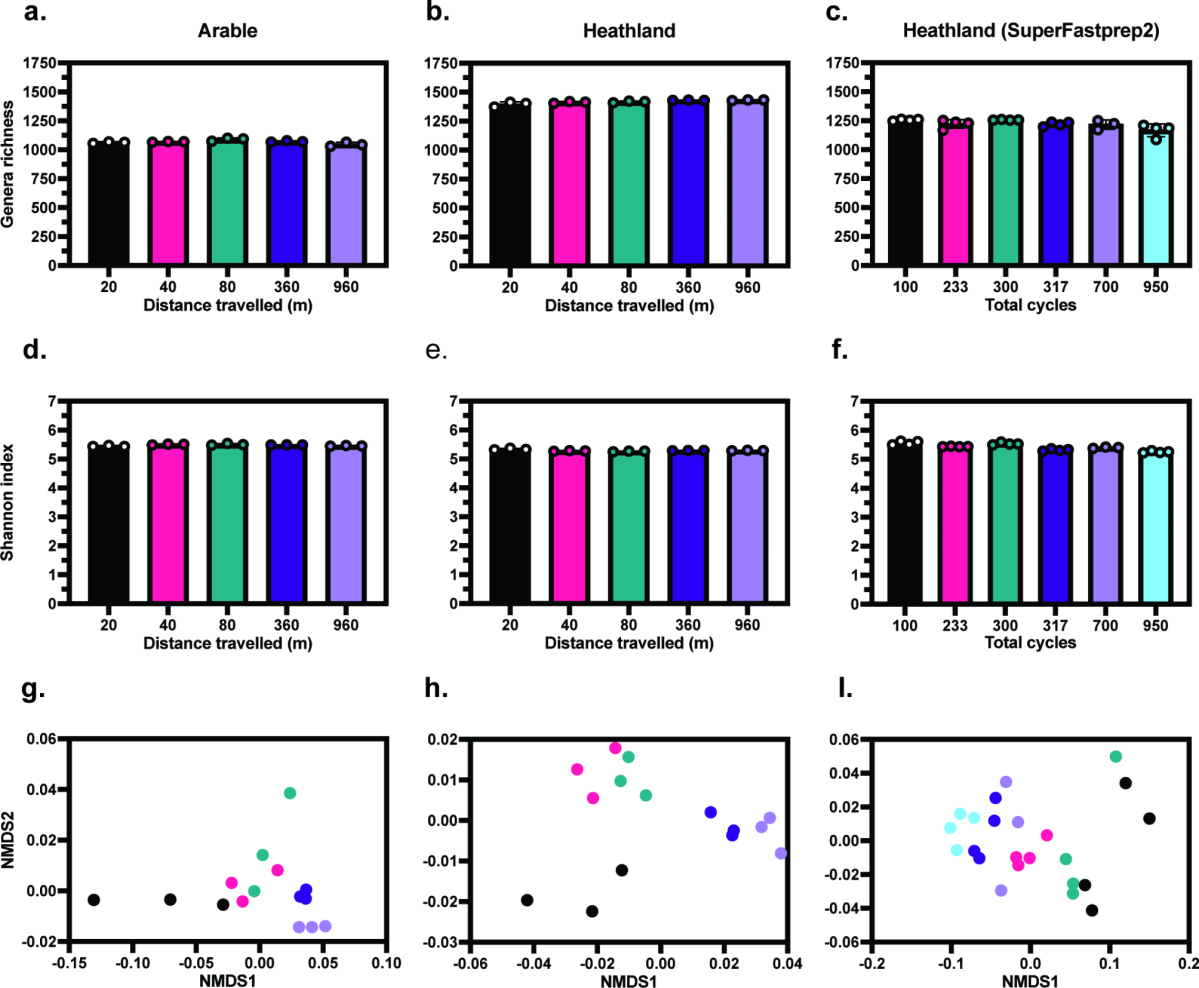
Ecological statistics for WGS. Genera richness (**A**–**C**) and Shannon diversity index (**D**–**F**) were calculated using genera counts after filtering of low count (< 10 reads) genera and rarefaction for all soil types and homogenisers. Non-metric multi-dimensional scaling was performed on Bray-Curtis distances calculated on rarefied datasets for each soil type and homogeniser (**G**, **H**,**I**). *n* = 3 (**A**,** B**, **D**,** E**,**G**,** H**); *n* = 4 (**C**, **F**, **I**) except for the 700 total cycles sample where a replicate was omitted due to failed sequencing preparation. A one-way ANOVA detected significant differences in genera richness between treatments (*p* < 0.05) for the arable soil (**A**). Kruskal-Wallis tests detected significant differences in genera richness between treatments (*p* < 0.05) for both Heathland soil DNA extracted using the FastPrep-24™ 5G (** B**) and SuperFastPrep-2™ (**C**). Significant differences in Shannon index were detected by Kruskal-Wallis test (*p* < 0.05) for the arable soil (**D**) and one-way ANOVA (*p* < 0.05) for both Heathland soil DNA extracted using the FastPrep-24™ 5G (** E**) and SuperFastPrep-2™ (** F**). PERMANOVA analysis was used to compare differences between heathland soil communities processed with FastPrep-24™ 5G and SuperFastPrep-2™ (G, I), no significant differences were detected (ADONIS2, *p* > 0.05).Error bars represent SD.

To assess the effect of homogenisation parameters on the observed microbiome, sequenced reads were taxonomically classified, then species richness, diversity and ecological distances between samples were compared for the observed genera in each sample (Fig. 4). Genera richness was calculated for each soil sample (Fig. 4A-C). While significant differences were observed between lysis conditions for each sample (one-way ANOVA, Kruskal-Wallis *p* < 0.05) absolute difference in genera richness between lysis conditions was small (~ 40 genera within both the arable and heathland samples), this was also true for the observed diversity index (Fig. 4D-F). Additionally, no significant differences in microbial community composition were observed between heathland soils processed using the FastPrep24 5G™ or SuperFastPrep-2™ when assessed by PERMANOVA (Fig. 4G-I, ADONIS2, *p* > 0.05). Saturation curves were performed on identified genera (Supplementary Fig. 6) indicating sufficient sequencing depth for community analysis for each sample. Relative abundance was quantified for each sample (Supplementary Fig. 7) with no significant differences detected between lysis conditions within any group (Kruskal-Wallis, *p* > 0.0.5). Bray-Curtis distance was calculated after rarefication of the data to ensure comparability between samples with differing read counts, non-metric multidimensional scaling was applied to the distance matrix (Fig. 4G-I). Microbial communities purified using the FastPrep24 5G™ from the heathland soil showed the clearest clustering by distance travelled (Fig. 4H), with more overlap observed in the arable soil and heathland soil lysed using the SuperFastPrep-2™.

### Metagenome assembly

**Fig. 5:**
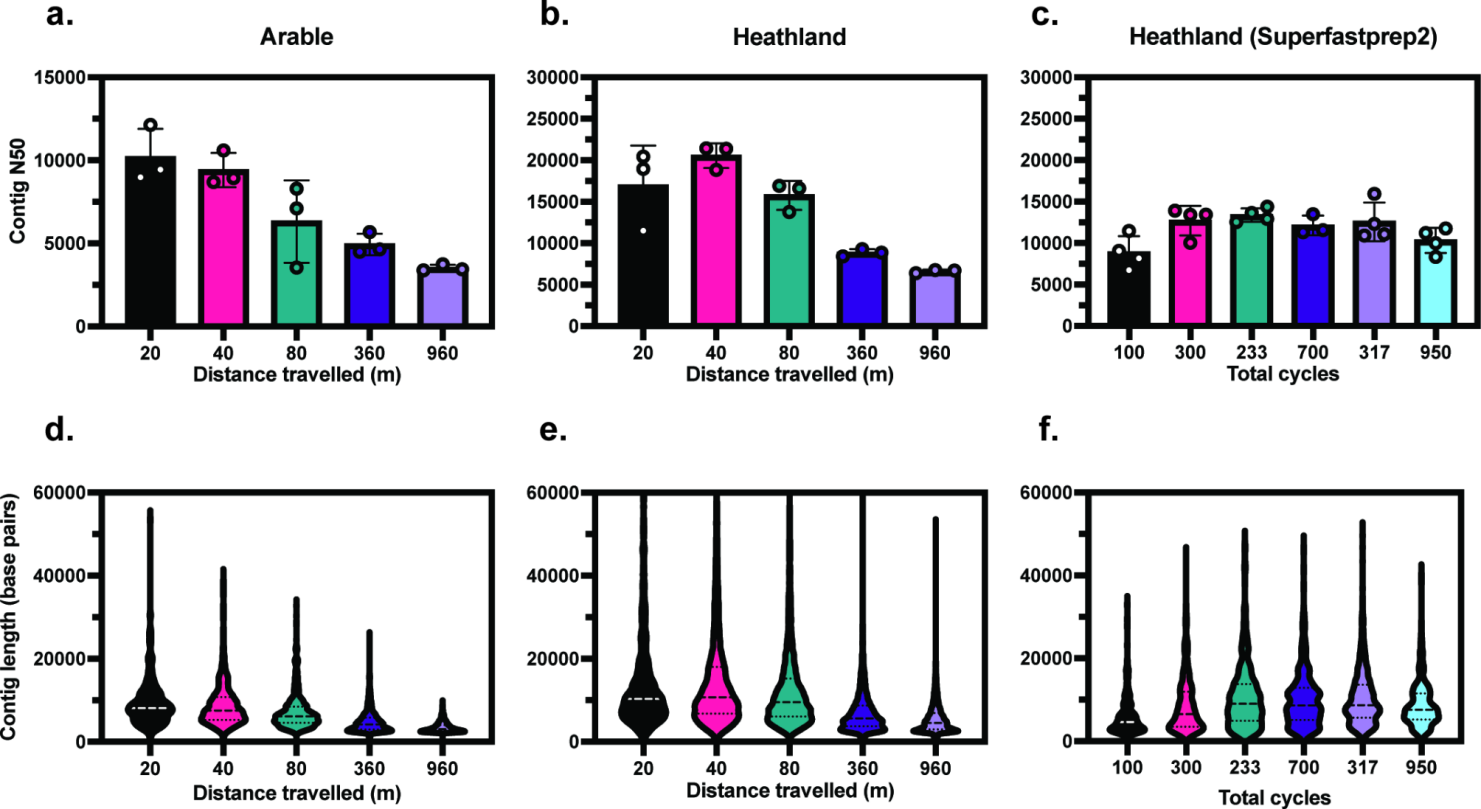
Metagenome assembly. Contig N50 (**A**–**C**) and contig length distribution (**D**–**F**) were quantified after metagenome assembly for each soil type and homogeniser. *n* = 3 (**A**, **B**,**D**, **E**); *n* = 4 (**C**, **F**), except for the 700 total cycles sample where a replicate was omitted due to failed sequencing preparation. A One-way ANOVA detected significant differences (*p* < 0.05) in contig N50 between treatments for the arable soil (**a**. Kruskal-Wallis tests detected significant differences in contig N50 (*p* < 0.05) between treatments for both Heathland soil DNA extracted using the FastPrep-24™ 5G (**B**) and SuperFastPrep-2™ (**C**). Error bars represent SD.

Metagenome assemblies were performed on DNA sequence data from both heathland and arable soils to assess the effect of increased DNA read length on assembled contig length. For both arable and heathland soils processed using the FastPrep24-5G™, the N50 of assembled contigs and the distribution of read lengths decreased with increasing distance travelled by the homogenisation tube (Fig. 5A, B,D, E), correlating with a reduction in the average read length for these data sets (Fig. 4A, B). Contig N50 and the distribution of contig lengths for the heathland soil were similar across all samples (Fig. 5C, F) using the SuperFastPrep-2™, and had a shorter total length when compared to the equivalent heathland soil datasets generated after lysis using the FastPrep24 5G™ (Fig. 5B, C).

## Discussion

Here we demonstrate that a DoE based approach, in which changes to DNA yield and fragment length were modelled based on the interaction and statistical contribution of homogenisation speed and time, was successful at improving the extraction of higher molecular weight DNA from soil. As mechanical lysis involves multiple continuously variable factors, a DoE based approach is well placed to optimise this process. We found that as few as 10 trial extractions can be used to screen for optimum lysis parameters which maximised DNA fragment length for multiple soil types using this method (Supplementary Fig. 3). The overlapping results for DNA yield and fragment length under different homogenisation regimes in our custom design indicate that a “one factor at a time” approach may have reached local optima and not fully realised the potential for extracting the longest DNA fragments possible. For soils within this study, the trade-off between DNA yield and mean fragment length was not a barrier to sequencing for extractions performed using the FastPrep24 5G™, as the lowest DNA yield observed was still sufficient for sequencing library preparation. However, for samples with low microbial load such as those taken from the subsoil^19^, performing a preliminary screen using the DoE approach outlined here may identify a lower limit for lysis parameters that yield DNA concentrations sufficient for effective sequencing. Custom protocols for the extraction of high molecular weight DNA from faecal samples have been developed by the community^20^. These custom protocols suggest mechanical lysis parameters similar to the common commercially available DNA extraction kits for use with soil after more gentle chemical lysis. DNA extraction methods that employ chemical and enzymatic lysis have been reported as purifying higher quality DNA than bead beating with the shearing effect of the beads cited as the probable cause. These methods could be applied to soil samples, however the throughput and reproducibility of working with a commercially available extraction kit in conjunction with fast mechanical lysis is preferable when considering the read lengths that are obtainable using our protocol. It is also important that all cells are efficiently lysed during DNA extraction, ensuring the full diversity of the soil sample is captured. Protocols that employ bead beating have been shown to more effectively capture the fungal diversity of a mixed sample than chemical/enzymatic methods^21^.

### Effect of homogeniser on microbial community

Homogenisation conditions made little difference to the alpha diversity or richness of the identified microbial communities for either soil tested. Differences between the relative abundance of genera in each sample were small with any differences occurring within the lowest abundance taxa. In this study most of the ecological diversity was found in the numerous low abundance taxa (< 1% relative abundance). No significant differences in relative abundance were detected for either the arable or heathland soil processed with the FastPrep24 5G™ even without filtering of low abundance taxa (Kruskal-Wallis, *p* > 0.99). Therefore, it was elected to combine this group into a single category for the purposes of representing the broad impact of lyses conditions on community representation. However, when filtering of low abundance taxa was not performed on samples processed using the SuperFastPrep2, those samples processed at 300 total cycles or fewer were found to be significantly different from samples processed at 317 total cycles and higher (Kruskal-Wallis and follow up Dunn’s test p = < 0.05). Therefore, it is not advisable to process samples using the SuperFastPrep2™ at a total cycles less that 317 as it may adversely impact the recovery of the microbial community as well as DNA concentration. Depending on the type of metagenomic study undertaken, rare taxa may be the target of downstream analysis for which the filtering performed here may exclude the desired data. Under these circumstances the level of homogenisation needed should be tailored to the analysis. For example, if assembly of MAGs for rare taxa is intended, increasing sample input into each extraction and increasing the depth of sequencing could retain the benefits of longer sequence reads while increasing coverage of the taxa of interest. For classification only studies, where fragment length maybe less important, rare taxa may be more easily captured with higher homogenisation speed and time.

### Community demography and functionality under differing lysis conditions

For analyses that requires functional gene recovery it is well documented that longer contig assemblies, which is improved through our framework, improve gene discovery^8^. However, functional traits in soil communities may differ less than the beta diversity of taxa may suggest^5^. For this reason, the inclusion of low abundance taxa in functional gene analysis may be important to capture the functional redundancy provided by the rarer genera. In this study both soil types had a low fungal: bacterial ratio (~ 1:50). Samples which exhibit a high proportion of taxa with tougher cell walls, such as fungi, may require longer homogenisation times to effectively purify DNA from the entire microbial community. Furthermore, there was little difference in the proportion of Gram positive and Gram negative bacteria identified between samples extracted within any lysis condition (Supplementary Fig. 7). This indicates that even the mildest lysis conditions tested could effectively extract DNA from both groups despite Gram positive bacteria possessing a tougher cell wall. This study focused on the impact of purifying larger DNA fragments on the microbial community within the soil. However, DNA purifications targeting organisms of a higher trophic level within the soil, such as nematodes and invertebrates, could also benefit from this approach potentially using alternative lysing matrices. Metal lysis tubes and bead mixes designed for processing particularly hard to lyse samples are available commercially (MP Biomedicals, USA). Using a harsher lysing matrix to recover DNA from higher trophic level organisms may result in greater DNA shearing from the easier to lyse organisms investigated here. Therefore, tailoring mechanical lysis to the requirements of a given study may be needed to balance community recovery and DNA fragment length.

### Sample specific considerations

Performing DNA extractions in the field has further potential to capture the true diversity of the soil microbiome. Here, we show in-field lysis can be performed with equivalent DNA yield and fragment length to the optimised lab-based protocol. As with the benchtop homogeniser, lower homogenisation speed and time correlated with a reduction in DNA yield and an increase in mean DNA fragment length. When comparing samples displaying similar DNA yield on the hand-held and benchtop extractions, mean fragment length was also similar. This indicates that, other than sample throughput, DNA extractions in the field can be comparable to the laboratory if the hand-held device is optimised to obtain a similar DNA yield. While the FastPrep24 5G™ homogeniser has a lower limit of 4 m s ^− 1^, hand-held homogenisation can be manually adjusted to much slower speeds, therefore allowing longer average fragment lengths to be achieved with the handheld device.

However, the trade-off between DNA integrity and yield was more pronounced when using the SuperFastPrep-2™ compared to the FastPrep24 5G ™, as DNA concentrations were below the recommended input requirements for library preparation, when using the ONT Native Barcoding Kit. Concentration by bead purification prior to library preparation may have impacted the size distribution and yield of purified DNA and hence contributed to the discrepancy between extracted DNA fragment length and sequence read length for these treatments. This could be addressed by altering other extraction parameters. As the input for library preparation here was limited by concentration rather than the total yield of DNA, reducing the final elution volume would increase DNA concentration. For samples with low microbial load, multiple replicate extractions could be performed, and the resulting DNA pooled to increase the final yield.

We found that improvements in mean DNA fragment length after extraction translated into longer sequenced reads and greater contiguous sequence length in subsequent metagenome assemblies. Contigs assembled from these longer reads could facilitate analysis that require high-quality genome assemblies, such as estimating mutation rates for soil microbe populations^22^, identifying complete biosynthetic gene clusters^23,24^ and the characterisation of metabolic pathways in microorganisms from extreme environments^25^. In addition, longer contigs perform better when resolving highly conserved and large homopolymer regions of the genome, leading to higher quality MAGs including complete bacterial genomes^10,26–28^.

Optimising homogenisation speed and time specifically to increase DNA fragment length significantly improved the length of subsequent sequence reads and the length of contiguous sequences after metagenome assembly. For a small number of scoping extractions, lysis parameters can be tuned for any soil type to ensure sufficient yield for sequencing with the longest DNA fragments possible extracted. From this study we recommend a homogenisation speed of 4 m s^− 1^ for a total of 10 s when using the FastPrep24-5G. These settings, while not giving the longest fragments found in the study, elicited comparable contig N50’s in resulting assemblies and ensured a DNA yield sufficient for the high sequencing depth required for high quality assemblies. Likewise, when using the handheld SuperFastPrep-2™, for speeds below 1900 cpm there was no benefit to fragment and subsequent read length while there was a significant drop in DNA yield, for these reasons we recommend a speed of 1900 cpm for 20–30 s to achieve DNA yields and fragment lengths comparable to extractions performed on the benchtop FastPrep24 5G™ with our recommended settings.

### Wider application of DOE framework for longer DNA fragments

The scope of soil research that could benefit from adopting long-read DNA sequencing is extensive, from studies elucidating gene clusters responsible for secondary metabolite dynamics^24^, to those investigating the impact of extreme weather events on microbial community function^29^ as well as the reconstruction of ancient ecosystems from sediment^30,31^. The method outlined here could help provide a framework for soil researchers to obtain the best quality DNA extractions tailored to their needs. Potentially leading to more complete metabolic pathway characterisation and MAG generation.

## Methods

### Sample collection and preparation

Soil was sampled from the top 10 cm of the soil profile from deciduous woodland (50.65239, -3.34743), an arable field (50.68772, -3.31217), a heathland (50.68523, −3.326668) and a permanent pasture (50.68029, -3.29261) within the Clinton Estate in Devon, England (Supplementary Fig. 1). Soil characteristics were determined from each land use type (Supplementary Table 3). Samples were processed by root sorting and sieving the bulk soil sample at 2 mm before being placed in storage at 4 ˚C. 20 g of each soil type was subsampled into individual pots with a perforated lid. Pots were incubated at 15 ˚C for seven days to ensure microbial activity increased after storage at 4˚C. DNA extractions were performed on 250 mg subsamples from each pot.

### DNA extraction, quantification and quality

All DNA extractions were performed using either the FastPrep-24 5G™ bead beater or SuperFastPrep-2™ hand-held homogeniser (MP Biomedicals, USA) in conjunction with the DNeasy PowerSoil Pro kit (Qiagen, Germany) with a target sample input of 250 mg. All steps after homogenisation were performed according to the manufacturer’s instructions. DNA was quantified using the Qubit Flex Fluorimeter in conjunction with the Qubit dsDNA HS assay kit (Thermo, USA). Mean fragment length was calculated using the Genomic DNA ScreenTape assay in conjunction with the 4200 TapeStation System (Agilent, UK). DNA fragments between 250 and 48,000 base pairs (bp) were used to determine mean fragment length.

### Custom DoE and statistical analysis

Custom experimental designs were generated using the DoE toolkit within the JMP Pro v16 software (Supplementary Table 1). DNA concentration and mean fragment length were specified as Y variables, and ‘maximise’ was set as the goal for each Y variable. Factors were parameterised based on process knowledge and recommendations given by the manufacturer. X variables included homogenisation speed, provided as metres per second (m s^− 1^) for the FastPrep24 5G™ and cycles per minute (cpm) for the SuperFastPrep-2™, total homogenisation time, and repeat homogenisations after an incubation period on ice for one minute. Cycles per minute of the SuperFastPrep-2™ was determined using a conversion chart detailed in the SuperFastPrep-2™ manual. All statistical modelling was performed using JMP Pro v16.

### Sequencing

All sequencing libraries were prepared using the Native Barcoding Kit 96 V14 (SQK-NBD.11496 ; Oxford Nanopore Technologies, UK) according to the manufacturer’s instructions. Samples with insufficient yields of DNA using the SuperFastPrep-2™ extraction kit were excluded. All replicates for extractions were performed using 100, 233 and 300 total cycles. A single replicate for both 317 and 700 total cycles were concentrated using a bead clean up by adding 120 µl of Mag-Bind TotalPure NGS beads (Omega Bio-Tek, USA) to 100 µl of DNA sample with a final elution of 25 µl. Prepared samples were sequenced using a R10.4.1 MinION flow cells coupled to a GridION (ONT, UK) and basecalled using Guppy v6.5.7 using the High accuracy model.

### Bioinformatics

Quality control of sequence reads was carried out during basecalling by Guppy v6.5.7. All reads that passed a Q score threshold of 7 then underwent adapter trimming using porechop v.0.2.4^32^. to remove any sequencing adapters from the reads that were not trimmed by Guppy v6.5.7 during basecalling. Adapter trimmed reads were taxonomically classified using centrifuge v.1.0.4^33^ against a custom database containing RefSeq complete genomes (November 2023) for all available bacteria, archaea, viruses, fungi, protozoa, plants, and vertebrate mammals. Count tables were generated using MEGAN v6.25.9 Ultimate edition^34^. Where fewer than 10 reads were classified as a particular genus for all samples, those genera were removed from the analysis. Relative abundance was then calculated after filtering by read count with further filtering performed by combining any genus with a relative abundance of < 1% for all samples into a group named “other”. Genera richness, Shannon-index, Bray-Curtis dissimilarity, Non-metric multidimensional scaling and ADONIS2 results were produced with the R package vegan v2.6-4^35^. PERMANOVA conducted with ADONIS2 used distance matrices generated using avgdist as input. The lowest total read counts of any sequenced sample were used as the sample size and Bray-Curtis as the dissimilarity index. GraphPad Prism v10.1.1 was used to perform Shapiro-Wilk normality tests, linear regression, One-way ANOVA and Kruskal-Wallis tests. Contigs were assembled using flye v2.9.3 (using the parameters *--meta --nano-hq*).

## Electronic supplementary material

Below is the link to the electronic supplementary material.

Supplementary Material 1

Supplementary Material 2

## Data Availability

The datasets generated during the current study are available in the NCBI Sequence Read Archive repository PRJNA1130143.
