# Supplementary material for: Statistical design approach enables optimised mechanical lysis for enhanced long-read soil metagenomics
Source: Sci Rep. 2024 Nov 22;14:28934. doi: 10.1038/s41598-024-80584-y (PMC11584900; doi:10.1038/s41598-024-80584-y)
Supplement: Supplementary file 2 — Supplementary Material 2 [file 41598_2024_80584_MOESM2_ESM.pdf]

Statistical design approach enables optimised mechanical lysis for enhanced long-read soil metagenomics - Supplementary Data.

Barber, Daniel G. [a], Child, Harry. [a],Joslin, Gabrielle. [a],Wierzbicki, Lucy. [a], Tennant, Richard K. [a*].

Abstract

Metagenomic analysis has enabled insights into soil community structure and dynamics. Long-read sequencing for metagenomics can enhance microbial ecology by improving taxonomic classification, genome assembly, and functional annotation. However, protocols for purifying high-molecular weight DNA from soil are not yet optimised. We used a statistical design of experiment approach to enhance mechanical lysis of soil samples, increasing the length of purified DNA fragments. Low energy input into mechanical lysis improved DNA integrity, resulting in longer sequenced reads. Our optimized settings of 4 m s^{-1} for 10 seconds increased fragment length by 70% compared to the manufacturer's recommendations. Longer reads from low intensity lysis produced longer contiguous sequences, improving a range of down-stream analyses. Importantly, there was minimal bias exhibited in the microbial community composition due to lysis efficiency variations. We therefore propose a framework for improving DNA fragment lengths from any soil type, improving soil science research with long-read sequencing.

Homogenisation Speed (m/s)	Cycles	Total Homogenisation Time (seconds)
4	2	120
6	1	120
4	2	90
6	2	60
8	1	90
8	1	120
4	1	60
6	2	120
4	1	30
8	2	30
8	2	30
6	1	30

Supplementary Table 1. Custom experimental design.

Experimental design generated using the custom tool in the DoE toolkit package of JMP Pro v16.

Homogenisation Speed (m s^{-1})	Time (seconds)	Input weight (mg)	Concentration (ng μl^{-1})	DNA yield (ng g^{-1})	Mean fragment length (bp)	nM	DIN	Purity A260:280
4	60	237	73.7	31.1	4956	72.7	5.8	1.83
4	60	253	77	30.4	4922	78.9	5.8	1.91
4	60	247	72.2	29.2	4388	148	5.6	1.75
4	30	226	59.4	26.3	5447	50.8	6.1	1.43
4	30	269	69.7	25.9	5390	59.7	6	1.85
4	30	266	70.6	26.5	5380	53.6	6	1.71
6	60	279	75.6	27.1				1.76
6	60	218	97	44.5	4265	130	5.5	1.82
6	60	224	75.2	33.6	4165	174	5.5	1.79
8	60	263	97.6	37.1	3483	107	5.1	1.73
8	60	232	90.4	39.0	3509	99	5.1	1.77
8	60	283	100	35.3	3593	85.2	5.1	1.76

Supplementary Table 2. Soil DNA extraction scoping trial

A conservative estimate of the effect size imparted by increasing homogenisation time on DNA fragment length was calculated as -459 bp from mean fragment length when homogenisation time was increased by 30 seconds. At this effect size the custom design showed good statistical power at 0.928. For DNA yield, the 5 ng μl^{-1} effect size determined from the scoping trial for both homogenisation speed and time coupled with the anticipated RMSE of 12 ng μl^{-1} resulted in poor statistical power of the custom design to detect the effect of either factor at 0.16 and 0.17 respectively. However, a difference of 5 ng μl^{-1} is not important for the optimisation of DNA extractions from soil, as the DNA concentrations extracted during the scoping trial across all experimental runs were sufficient for multiple sequencing library preparations (50-90 ng μl^{-1}). An effect size of 20 ng μl^{-1} would represent a more relevant effect size as this could yield several more sequencing library preparations. The statistical power of the custom design increased significantly to 0.96 for homogenisation speed and 0.97 for homogenisation time with an effect size of 20 ng μl^{-1} for DNA yield.

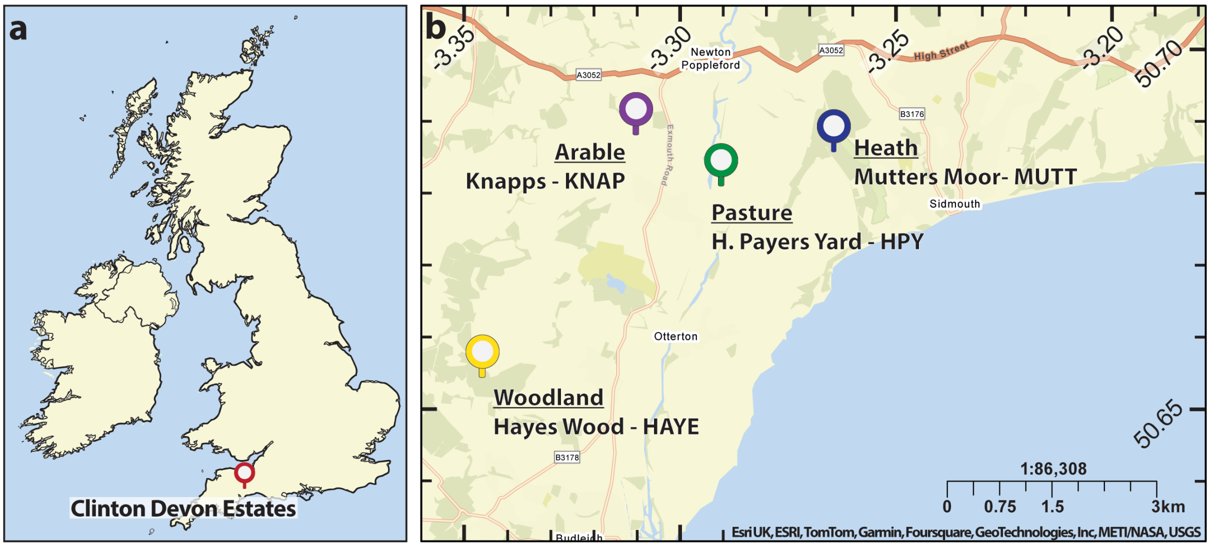

Supplementary Figure 1: Map of Sampling Locations

(a) Location of Clinton Devon Estates, UK. (b) location of Woodland (yellow), Arable (purple), Pasture (green) and Heathland (blue)

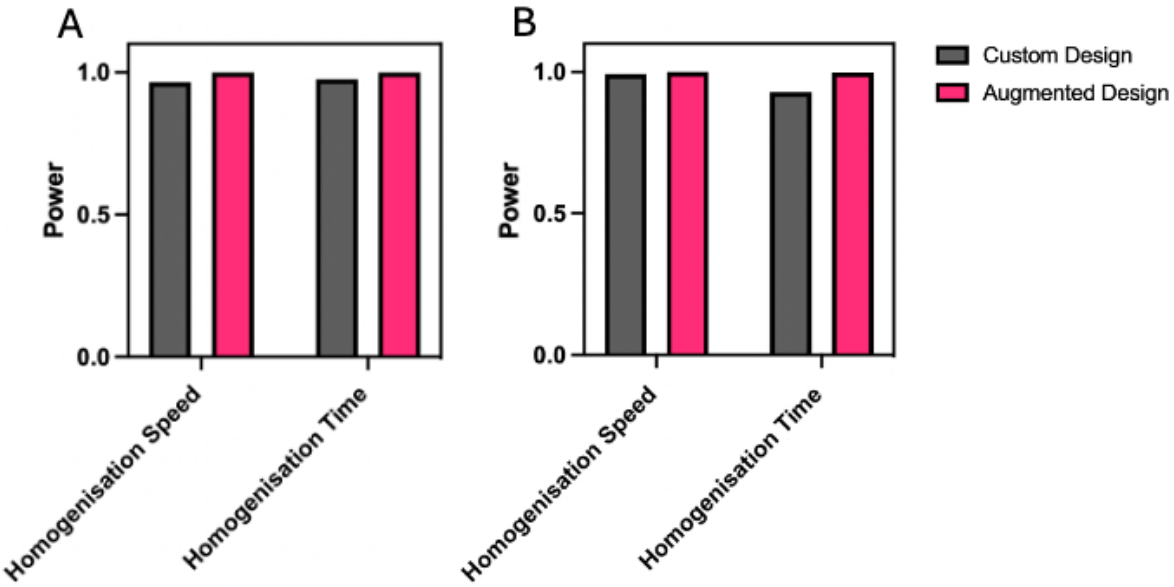

Supplementary Figure 2: Power Analysis

A. Statistical power of the JMP custom experimental design vs augmented custom design for DNA concentration with an RMSE of 12 ng ul^{-1} and anticipated coefficient of 20 ng ul^{-1}. B. Statistical power of the JMP custom experimental design vs augmented custom design for fragment size with an RMSE of 318 bp, the maximum error observed in the scoping trial, and anticipated coefficient -459 bp for time and -615 bp for speed.

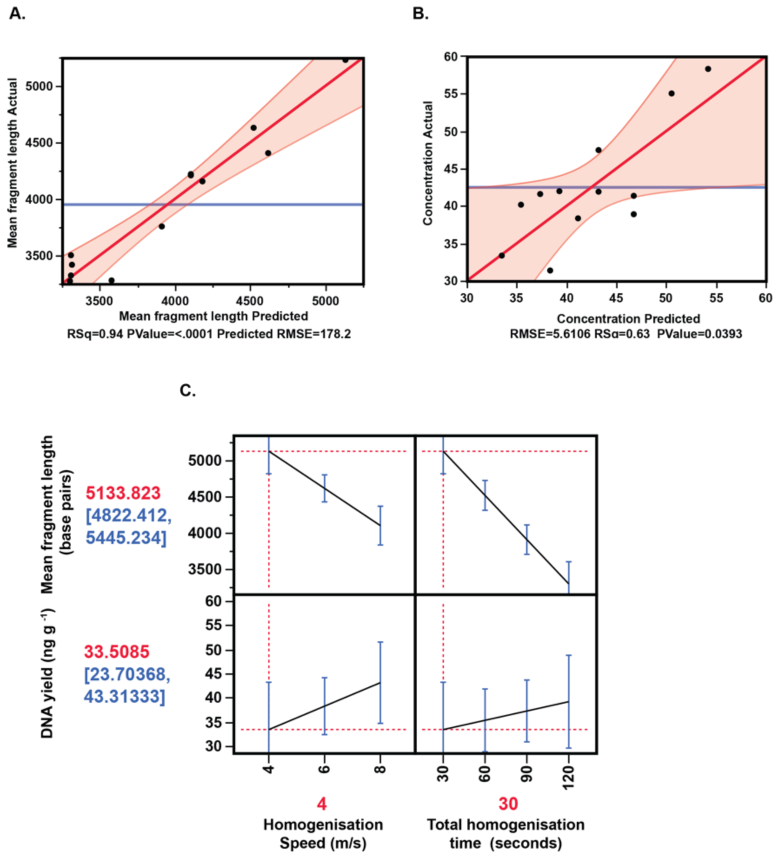

Supplementary Figure 3: Evaluation of least squares regression model

Least squares regression model performance in predicting mean fragment length, R^2 0.94, $p < 0.001$, (A) and DNA yield, R^2 0.63, $p < 0.05$ (B) were determined after removal of non-significant factors from the model. The output of the prediction profiler tool (C) showed further runs could provide further increases in mean fragment length while maintaining DNA yield at adequate concentrations for sequencing. Red shaded area represents 95% confidence interval (A,B). Error bars represent standard error (C).

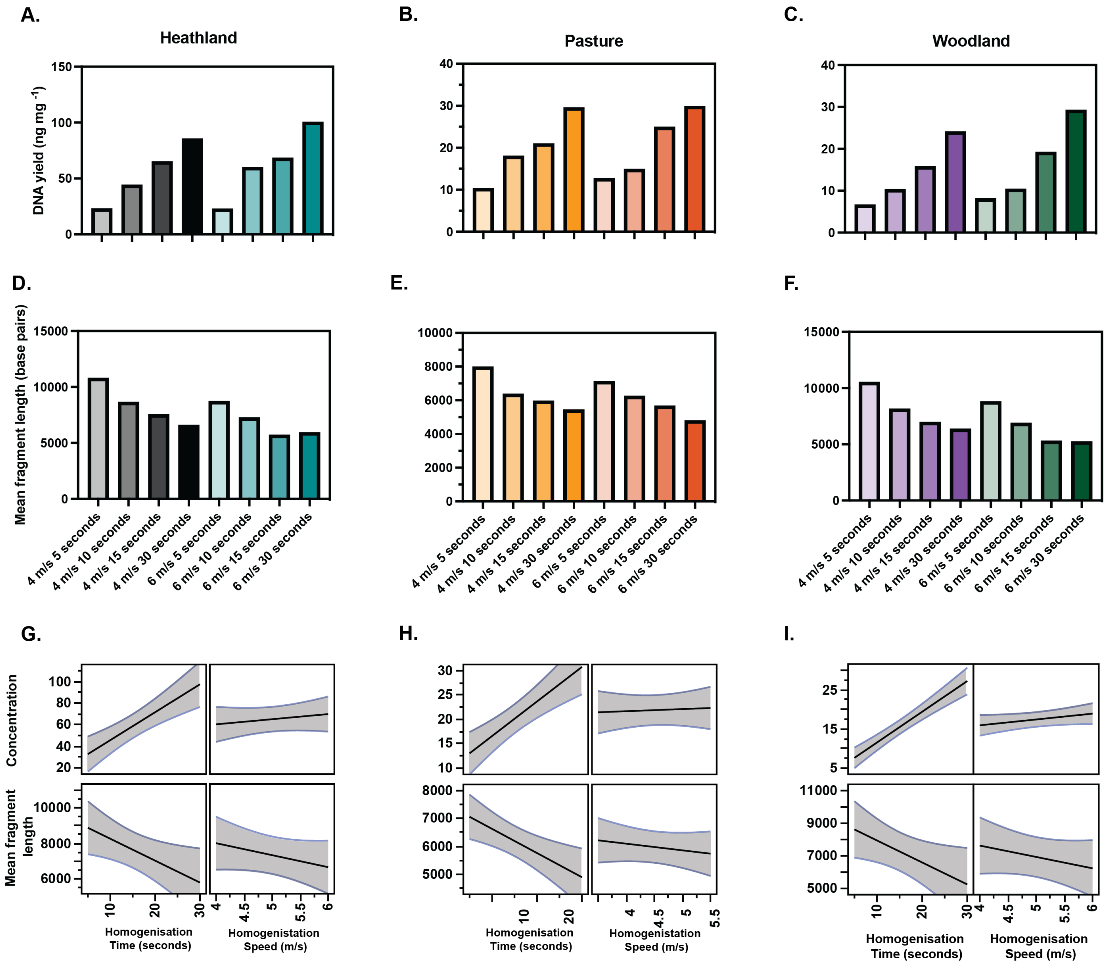

Supplementary Figure 4: Full factorial on different soil types.

Single replicate DNA extractions on three different soil types were performed using a full factorial design generated using the DoE toolkit in JMP 16 v2. DNA yield (A-C) and mean fragment length (D-F) were determined for each soil type. The prediction profiler output within JMP 16 v2 show trends in DNA yield and mean fragment length in response to changes in homogenisation speed and time (G-I).

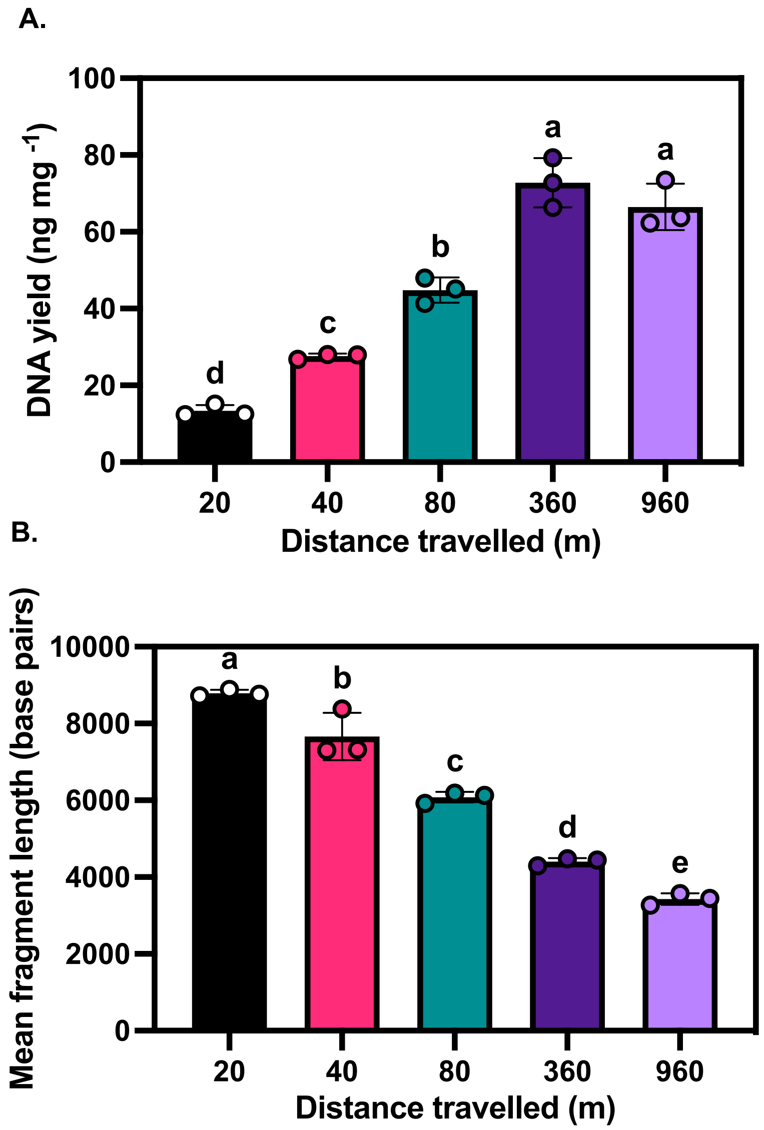

Supplementary Figure 5. Heathland soil DNA extractions.

DNA extractions were performed on Heathland soil samples using the FastPrep24 5G with equivalent homogenisation parameters to arable samples chosen for sequencing. Compact letter display indicates the results of a One-Way ANOVA with Tukey's multiple comparisons test whereby by groups with common letters were not found to be significantly different. n = 3, error bars represent SD.

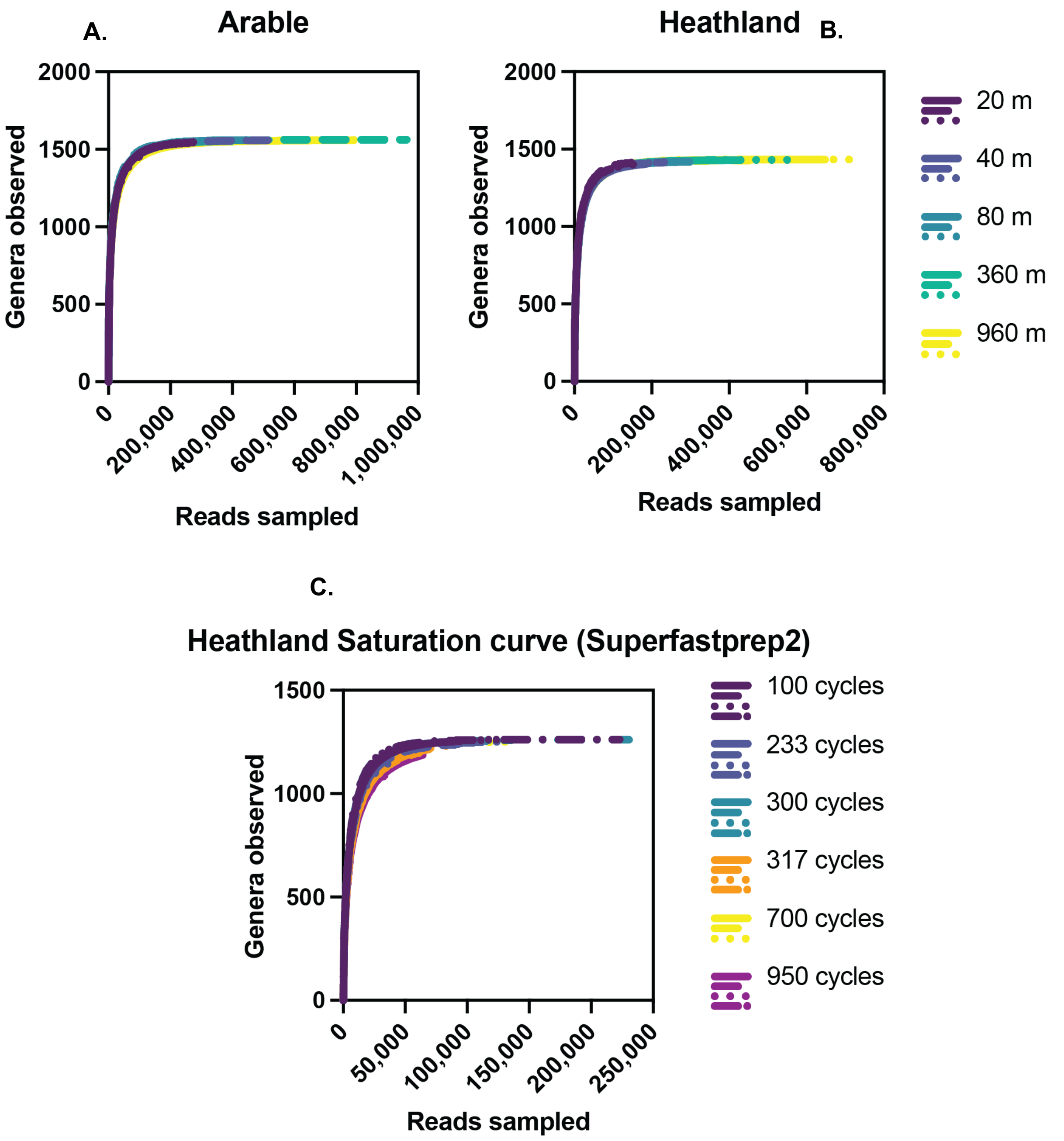

Supplementary Figure 6. Saturation curves.

Rarefaction was performed on genus count data for each of the sequenced samples and number of genera recorded after each sampling (A,B,C). Line quality represents replicate samples.

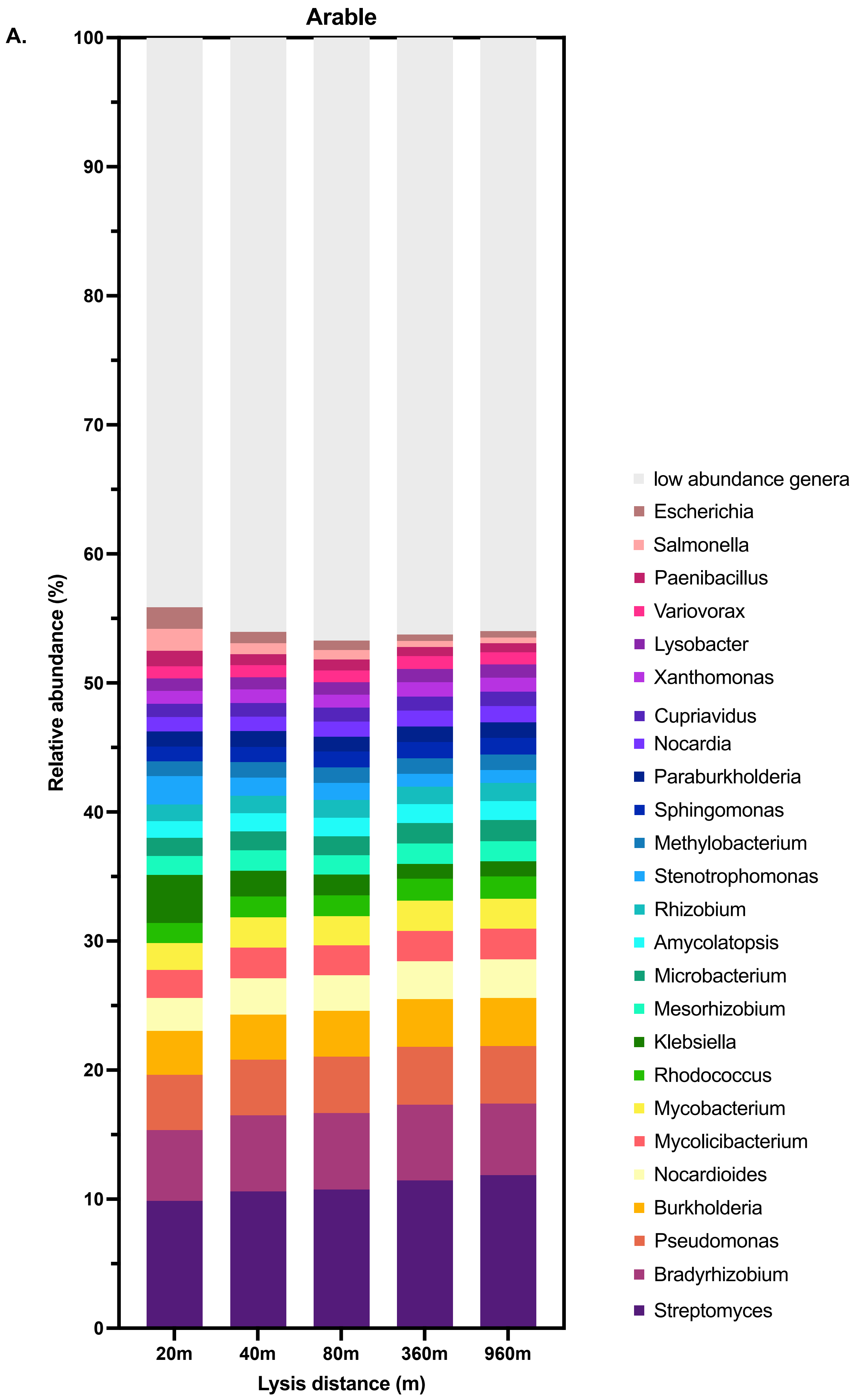
A.
Arable
Relative abundance (%)
0
10
20
30
40
50
60
70
80
90
100
20m
40m
80m
360m
960m
Lysis distance (m)
low abundance genera
Escherichia
Salmonella
Paenibacillus
Variovorax
Lysobacter
Xanthomonas
Cupriavidus
Nocardia
Paraburkholderia
Sphingomonas
Methylobacterium
Stenotrophomonas
Rhizobium
Amycolatopsis
Microbacterium
Mesorhizobium
Klebsiella
Rhodococcus
Mycobacterium
Mycolicibacterium
Nocardioides
Burkholderia
Pseudomonas
Bradyrhizobium
Streptomyces

B.

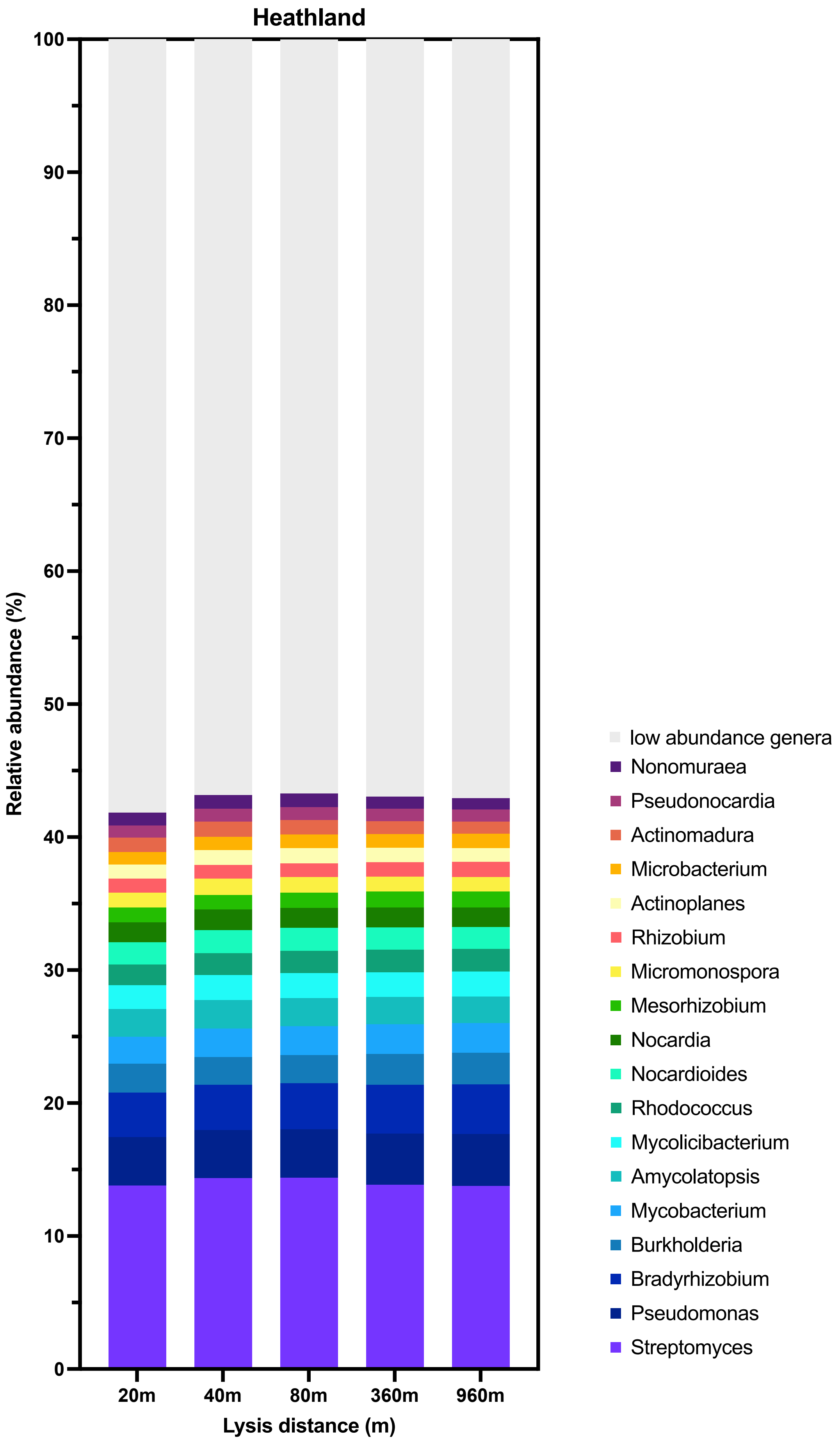

Heathland
Relative abundance (%)
100
90
80
70
60
50
40
30
20
10
0
20m
40m
80m
360m
960m
Lysis distance (m)
low abundance genera
Nonomuraea
Pseudonocardia
Actinomadura
Microbacterium
Actinoplanes
Rhizobium
Micromonospora
Mesorhizobium
Nocardia
Nocardioides
Rhodococcus
Mycolicibacterium
Amycolatopsis
Mycobacterium
Burkholderia
Bradyrhizobium
Pseudomonas
Streptomyces

C.

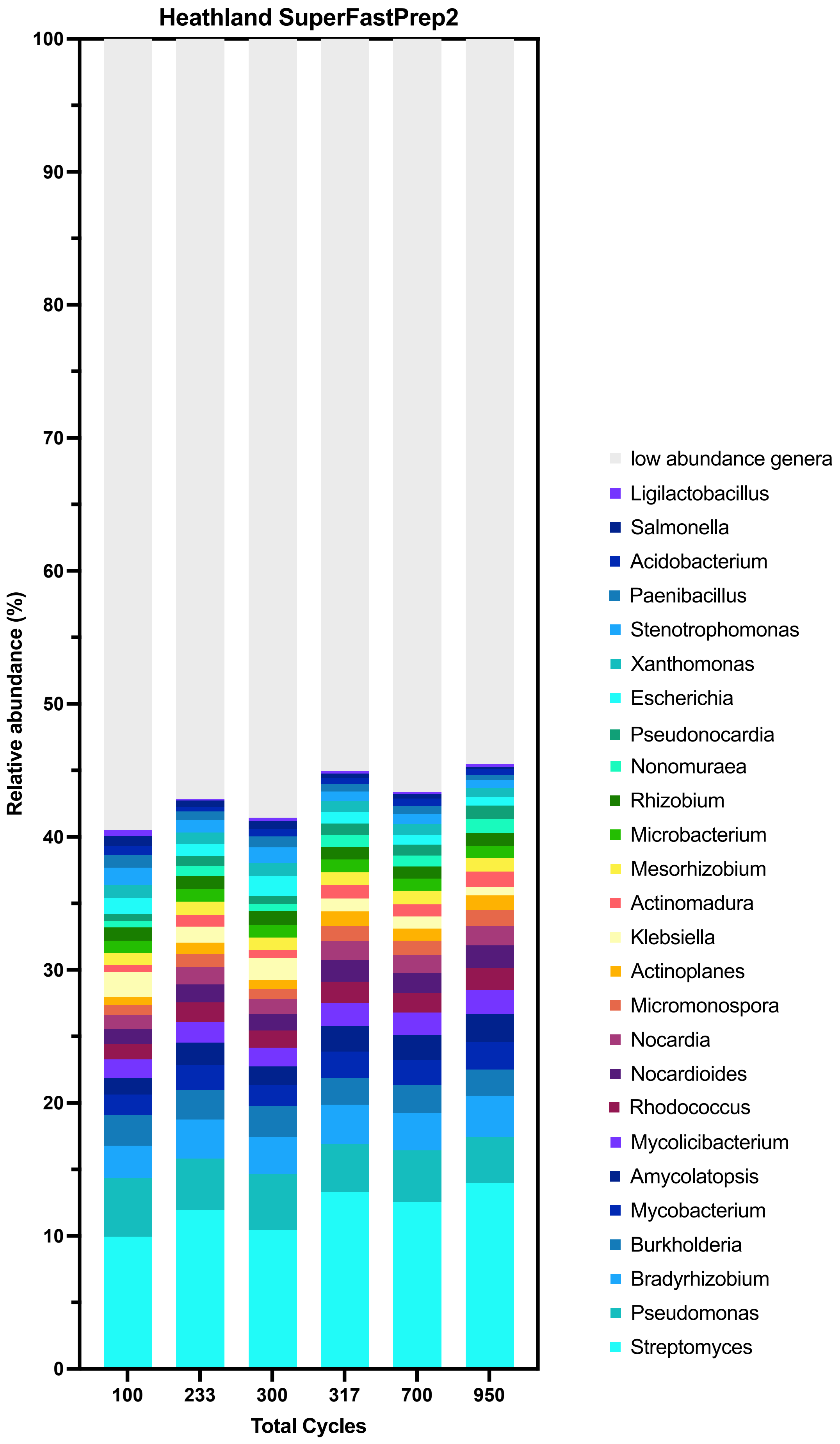

Heathland SuperFastPrep2
Relative abundance (%)
0
10
20
30
40
50
60
70
80
90
100
100
233
300
317
700
950
Total Cycles
low abundance genera
Ligilactobacillus
Salmonella
Acidobacterium
Paenibacillus
Stenotrophomonas
Xanthomonas
Escherichia
Pseudonocardia
Nonomuraea
Rhizobium
Microbacterium
Mesorhizobium
Actinomadura
Klebsiella
Actinoplanes
Micromonospora
Nocardia
Nocardioides
Rhodococcus
Mycolicibacterium
Amycolatopsis
Mycobacterium
Burkholderia
Bradyrhizobium
Pseudomonas
Streptomyces

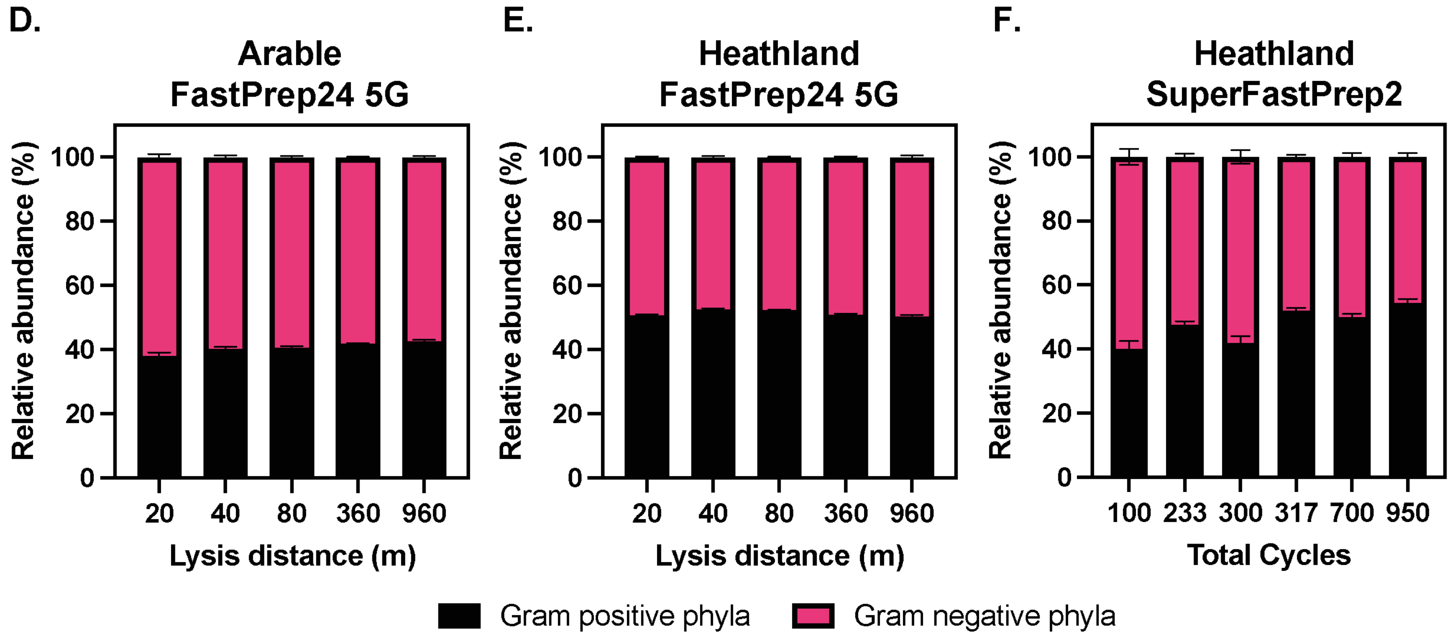

Supplementary Figure 7: Relative abundance of arable and heathland soil communities.

Relative abundance of genera classified within the arable and heathland soils soil samples after filtering of low count (< 10) taxa and after combining all taxa with < 1% relative abundance into a single low abundance group designated other (A,B,C). Figure legends indicate the 12 most abundant genera. Each bar represents the mean relative abundance (A,B. n =3) of metagenomes generated using the FastPrep24 5G and SuperFastPrep2 (C, n = 4). Proportion of bacterial gram positive and gram negative bacteria classified in each sample (D,E,F) determined by reads classified as Actinobacteria and Proteobacteria respectively.

Soil type	Dominant plant species	Sampling depth (cm)	Soil pH	Organic matter (%)	Carbon content (%)	Field capacity (%)	NATMAP Soil Classification
Arable	Spring Triticale	0-10	5.91	3.66	2.12	26.51	Deep Loam
Heathland	Common heather (Calluna vulgaris)	0-10	3.87	52.57	30.49	48.34	Loam over red sandstone
Pasture	Mixed grass (dominant Ryegrass Lolium perenne)	0-10	6.17	5.27	3.04	35.41	Seasonally wet deep silty
Woodland	Oak (Quercus robur) and Beech (Fagus sylvatica)	0-10	3.5	12.95	7.51	40.88	Deep Loam

Supplementary Table 3. Soil characteristics for Clinton Estate samples
